# Interactive effects of rhizospheric soil microbes and litter on the growth of the invasive hyperaccumulator *Bidens pilosa* in cadmium-contaminated soil

**DOI:** 10.3389/fpls.2024.1507089

**Published:** 2024-12-12

**Authors:** Xue Wang, Wei-Long Zheng, Chun-Lan Wu, Jing-Jing Han, Yu-Peng Xiang, Ming-Lang Yang, Peng He, Fei-Hai Yu, Mai-He Li

**Affiliations:** ^1^ Institute of Wetland Ecology & Clone Ecology/Zhejiang Provincial Key Laboratory of Plant Evolutionary Ecology and Conservation, Taizhou University, Taizhou, Zhejiang, China; ^2^ Tianjin Key Laboratory of Animal and Plant Resistance, College of Life Sciences, Tianjin Normal University, Tianjin, China; ^3^ Forest Dynamics, Swiss Federal Institute for Forest, Snow and Landscape Research WSL, Birmensdorf, Switzerland

**Keywords:** *Bidens pilosa*, Cd, fungi, phytoremediation, plant-soil feedback

## Abstract

Both rhizospheric soil microbes and shoot litter input can have profound effects on plant performance; however, their interactive effects on plants in Cd-contaminated soils remain poorly understood. We grew an invasive hyperaccumulator, *Bidens pilosa*, in sterilized and unsterilized rhizosphere soil without litter or with a low (0.2%, dry weight ratio) or a high amount (1%) of litter from *B. pilosa* in soil with low (5 mg kg^−1^) or high (10 mg kg^−1^) concentrations of Cd. The total, shoot, and root biomass of *B. pilosa* increased significantly with litter addition, by an average of 27%, 28%, and 20%, respectively. The biomass of *B. pilosa* was significantly lower in unsterilized rhizosphere soil than in sterilized rhizosphere soil, decreasing by 19% for total, 18% for shoot, and 24% for root, respectively. Furthermore, the effects of different litter amounts (0.2% vs. 1%) on biomass did not vary in sterilized rhizosphere soils but significantly varied in unsterilized rhizosphere soils, showing that the biomass was significantly lower with 1% litter addition than with 0.2% litter addition in unsterilized rhizosphere soils, decreasing by 28% for total, 29% for shoot, and 21% for root, respectively. Tissue Cd concentrations were significantly higher in highly Cd-contaminated soils (+75% for shoot and +51% for root) than in low Cd-contaminated soils; however, higher tissue Cd concentrations did not cause a significant decrease in the biomass of *B. pilosa*. Soil fungal communities, particularly the dominant phyla, Ascomycota and Basidiomycota, play crucial roles in modulating the effects of rhizosphere soil microbes and litter on the growth of *B. pilosa*. Our results suggest that rhizosphere soil microbes and litter interact and affect the growth of *B. pilosa*: litter addition promoted growth by increasing the abundance of saprotrophs (especially Basidiomycota) and decreasing Cd accumulation in plant tissues, and rhizosphere soil inhibition was associated with a decreased abundance of Basidiomycota. Our findings highlight the importance of the interactive effects of rhizospheric soil microbes and litter on plant growth in Cd-contaminated soils.

## Introduction

The frequency of plants exposed to soils contaminated with heavy metals has increased as a result of human activities such as industrial discharges, mining and smelting, and agricultural pollution (Mazurek et al., 2017; Palansooriya et al., 2020). Cadmium (Cd) is one of the most toxic heavy metals and can cause significant damage to plants (Wang et al., 2024; Zheng et al., 2023). For example, Cd toxicity can inhibit root elongation, photosynthesis, stomatal conductance, and enzyme activities in plants (Haider et al., 2021; Li et al., 2023a). Rhizospheric soil microbes and plant litter can influence plants through plant–soil feedback (De Long et al., 2022; Sun et al., 2022; Zotti et al., 2023). However, the effects of rhizospheric soil microbes and litter on the growth performance of plants in Cd-contaminated soils are understudied.

Plants have the ability to modify biotic and abiotic soil environments through the direct effects of the rhizosphere and indirect effects of litter input as they grow in the soil (Aldorfova et al., 2022; He et al., 2023; Zhang et al., 2019). These modifications can lead to plant–soil feedback (PSF), whereby the performance of the same or different plant species is influenced by the modified soil (Bever et al., 1997; van der Putten et al., 2013). PSF can result in either positive or negative effects when the performance of conspecifics is enhanced or inhibited (Bennett et al., 2017; Jing et al., 2022; Teste et al., 2017). For example, negative PSF would prevail if host-specific pathogens accumulate in the rhizosphere, whereas the species having higher mycorrhizal colonization would result in mostly positive PSF (Bennett and Klironomos, 2019; Semchenko et al., 2022). Therefore, the intensity and direction of PSF play a crucial role in shaping plant growth, survival, and distribution.

Three main groups of soil biota contribute to PSF: enemies (pathogens and root-feeding insects), mutualistic symbionts (mycorrhizal fungi and rhizobia), and decomposers (Friman et al., 2021; Idbella et al., 2024; Kadowaki, 2024). Diverse communities of soil microorganisms and invertebrates that accumulate in the rhizosphere are expected to influence PSF, with negative and positive effects (Kadowaki, 2024). As for soil microorganisms, fungi, especially soil pathogenic or mycorrhizal fungi, can play important roles in regulating PSF (van der Putten et al., 2013). For example, rhizosphere-induced negative PSF effects occur when pathogens dominate the rhizosphere, thereby inhibiting plant growth (Bezemer et al., 2013). Conversely, rhizosphere-induced positive PSF effects occur when mutualistic symbionts, such as arbuscular mycorrhizal fungi (AMF), dominate the rhizosphere and promote plant growth (Garcia-Parisi and Omacini, 2017; Wang et al., 2019).

Shoot litter is also expected to have varying effects on PSF, ranging from negative to positive (Eppinga and Molofsky, 2013; Zotti et al., 2023). Fungi play an important role in regulating the litter-induced PSF. For example, some fungi, such as the two fungal phyla Ascomycota and Basidiomycota, would be very active during litter decomposition, because the two phyla include many saprotrophic members (Voriskova and Baldrian, 2013; Zhang et al., 2018). Negative PSF effects can occur through increased pathogen abundance and autotoxicity effects owing to the release of self-DNA and allelopathic compounds from conspecific litter (Idbella et al., 2024; Mazzoleni et al., 2015). Low-quality litter and slow decomposition, characterized by high concentrations of lignin and cellulose, can also lead to negative PSF effects (Ehrenfeld et al., 2005). Conversely, nutrient-rich litter inputs can enhance the availability of soil nutrients; for example, the available N and P increased from tundra to forest soils corresponding to the increase in nutrient contents in foliage of trees along the same direction (Fetzer et al., 2024), thus contributing to positive PSF and promoting plant growth (Eppinga and Molofsky, 2013; Sun et al., 2022). Although many studies have examined the relationships between litter-induced PSF and plant performance, little is known about the interactive effects of litter and rhizospheric soil microbes on the growth performance of plants growing in heavy metal-contaminated soils.

To investigate the interactive effects of rhizospheric soil microbes and litter on the growth performance of plants in Cd-contaminated soil, we conducted a pot experiment using the invasive plant species *Bidens pilosa* L., which is known to be a Cd hyperaccumulator (Sun et al., 2009; Zhang et al., 2021). We aimed to address the following questions: 1) How do rhizospheric soil microbes and shoot litter influence the growth performance of *B. pilosa* in Cd-contaminated soil? 2) Are there interactive effects between rhizospheric soil microbes and shoot litter on the growth performance of *B. pilosa* in Cd-contaminated soil?

## Materials and methods

### The collection of seeds, shoot litter, and rhizosphere soil of *Bidens pilosa*

Healthy seeds and withered shoots of *B. pilosa* were collected from a local population in Taizhou, Zhejiang Province, China, at the end of the growing season in November 2022. The withered shoots were air-dried and ground to pass through a 0.25-mm sieve. The ground shoots and seeds were stored at 4°C for later use. The withered shoots contained 0.47 ± 0.12 mg kg^−1^ Cd, 3.08 ± 0.33 mg g^−1^ nitrogen (N), and 1.53 ± 0.12 mg g^−1^ P. In August 2023, *B. pilosa* seeds were sown in plastic containers. In September 2023, rhizosphere soil was collected from the same plant population from which the litter and seeds were collected. The collection involved removing the roots of *B. pilosa* from the soil, collecting the soil attached to the roots, and storing it in plastic bags. The collected rhizosphere soil was immediately transported to the laboratory for use in experiments.

### Experimental design

**Figure 1:**
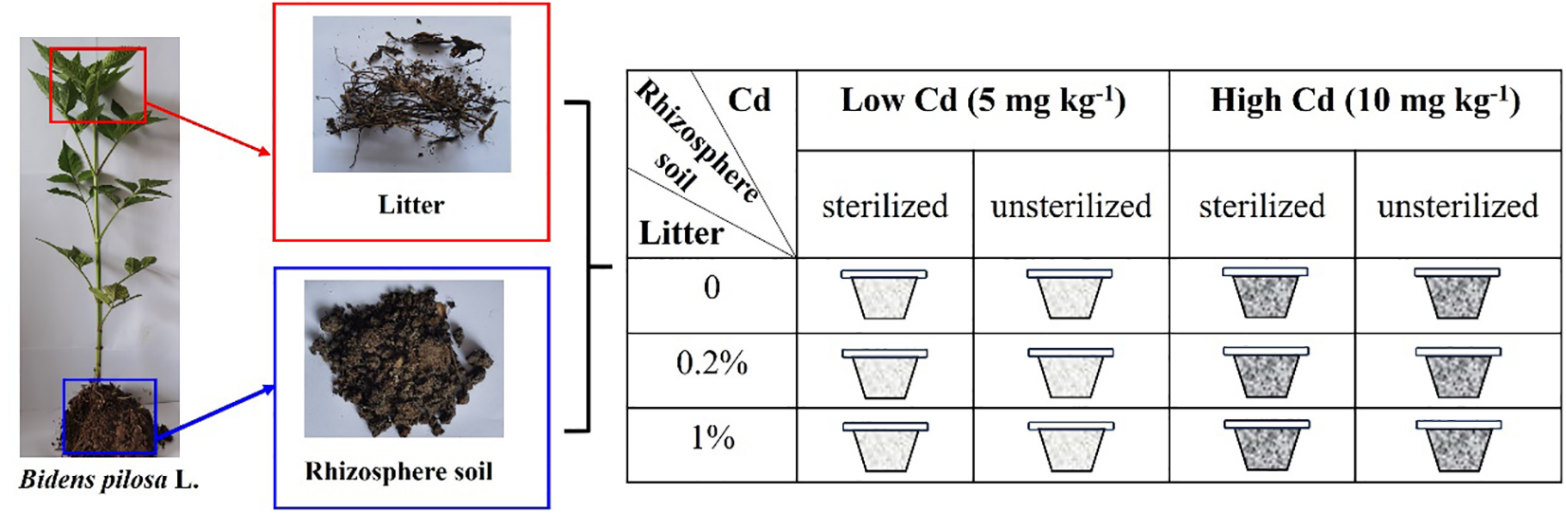
Graphical illustration of the experimental design. The experiment consisted of three levels of litter amounts (0%, 0.2%, and 1% of the total weight of the substrate), two levels of rhizospheric soil microbe treatments (sterilized and unsterilized), and two levels of soil Cd concentration (5 mg kg^−1^ and 10 mg kg^−1^).

The experiment comprised three levels of litter treatments (0%, 0.2%, and 1% litter), two rhizospheric soil microbe treatments (sterilized and unsterilized soil), and two Cd-contaminated soil treatments (low: 5 mg kg^−1^ vs. high: 10 mg kg^−1^) (**Figure 1**). Each treatment consisted of six replicates, resulting in 72 pots in total. In September 2023, the field soil was collected from a hill in Taizhou City. The field soil contained 0.54 ± 0.09 mg kg^−1^ Cd, 92.05 ± 15.65 mg kg^−1^ N, and 211.88 ± 28.38 mg kg^−1^ P. The field soil was air-dried and sieved through a 2-cm mesh to remove large stones and roots, and it was used to fill the 72 pots. Before filling the pots, the field soil was sterilized by autoclaving at 121°C for 120 min, and the pots were surface-sterilized with 75% ethanol. The rhizosphere soils were divided into two equal parts. One part was sterilized by autoclaving, while the other part remained unsterilized. The litter and rhizosphere soil treatments included sterilized soil, unsterilized soil, sterilized soil with 0.2% litter, unsterilized soil with 0.2% litter, sterilized soil with 1% litter, and unsterilized soil with 1% litter. Twelve pots were used for each treatment. For the sterilized soil treatment, 0.25 kg of field soil was added to each pot, followed by 0.05 kg of sterilized rhizosphere soil (equivalent to 10% of the total weight of the substrate), and 0.2 kg of field soil. Similarly, for the unsterilized soil treatment, 0.25 kg of field soil was added to each pot, followed by 0.05 kg of unsterilized rhizosphere soil and 0.2 kg of field soil. For the sterilized soil + 0.2% litter treatment, 0.25 kg of field soil was added to each pot, followed by 0.05 kg of sterilized rhizosphere soil, and a mixture of 0.199 kg of field soil and 0.001 kg of litter (equivalent to 0.2% of the total weight of the substrate). Similarly, for the unsterilized soil + 0.2% litter treatment, 0.25 kg of field soil was added to each pot, followed by 0.05 kg of unsterilized rhizosphere soil, and a mixture of 0.199 kg of field soil and 0.001 kg of litter. For the sterilized soil + 1% litter treatment, 12 pots were filled with 0.25 kg of field soil, followed by 0.05 kg of sterilized rhizosphere soil, and a mixture of 0.195 kg of field soil and 0.005 kg of litter (equivalent to 1% of the total weight of the substrate). Similarly, for the unsterilized soil + 1% litter treatment, 12 pots were filled with 0.25 kg of field soil, followed by 0.05 kg of unsterilized rhizosphere soil, and a mixture of 0.195 kg of field soil and 0.005 kg of litter. Then, each of the six treatments was divided into two equal parts, with 6 pots receiving 50 mL of a 50 mg L^−1^ CdCl_2_·2.5H_2_O solution and the other 6 pots receiving 50 mL of a 0.1 g L^−1^ CdCl_2_·2.5H_2_O solution. This division aimed to create two levels of Cd-contaminated soil treatments, corresponding to concentrations of 5 mg kg^−1^ and 10 mg kg^−1^. The 10 mg kg^−1^ Cd represented the highest level found in Cd-contaminated soil in Taizhou City (Wu et al., 2019), and half of the highest value was selected as a lower value. Two weeks after the application of the CdCl_2_·2.5H_2_O solution, one seedling of *B. pilosa* was planted in each pot. Dead seedlings were replaced during the first week of the experiment. All pots were watered every 2 days.

### Harvest

After 12 weeks following transplantation, the shoots and roots of *B. pilosa* in all pots were harvested. The shoots and cleaned roots were then oven-dried at 65°C for 72 h before being weighed. Subsequently, the shoots and roots were ground to pass through a 0.25-mm mesh for Cd analysis. Soil samples were also collected for the analysis of soil parameters.

### Cd and N analyses

To determine the total Cd concentrations in both the plant tissues and soils, an inductively coupled plasma mass spectrometry (ICP-MS) instrument (NexION 2000B, PerkinElmer, USA) was utilized. Prior to analysis, both the tissues and soils were digested using a mixture of sulfuric and perchloric acid in a ratio of 10:1. Additionally, total soil N was measured using an AutoAnalyzer 3 instrument (Bran & Luebbe, Norderstedt, Germany) after digestion with the same sulfuric and perchloric acid mixture in a ratio of 10:1.

### Soil microbial community analysis

Library preparation, sequencing, and bioinformatic analysis of the soil microbial community were conducted by Novogene Co., Ltd. (Beijing, China). Total DNA was extracted from each soil sample (0.5 g) using the Magnetic Soil and Stool DNA Kit (Tiangen, Beijing, China) following the manufacturer’s instructions. After determining the quality of each sample, a distinct region of the ITS gene was amplified by PCR using specific primers: ITS1-1F-F-GCATCGATGAAGAACGCAGC and ITS1-1F-R-TCCTCCGCTTATTGATATGC. The PCR reactions were carried out with 15 µL of Phusion^®^ High-Fidelity PCR Master Mix (New England Biolabs [Beijing] Co., Ltd.), 0.2 µM of forward and reverse primers, and 10 ng of template DNA. The PCR reaction condition was shown as follows: initial denaturation at 98°C for 1 min, followed by 30 cycles (denaturation at 98°C for 10 s, annealing at 50°C for 30 s, and elongation at 72°C for 30 s) and final extension at 72°C for 5 min. After amplification, the PCR products were purified using magnetic beads and mixed in the proportions required for sequencing. The libraries were generated with the NEBNext^®^ Ultra™ II DNA Library Prep Kit (New England Biolabs [Beijing] Co., Ltd.), then pooled and sequenced on an Illumina NovaSeq platform (Illumina, San Diego, California, USA), according to effective library concentration and data amount required. After sample splitting, the paired-end reads were merged using FLASH (V1.2.11, http://ccb.jhu.edu/software/FLASH/) (Magoč and Salzberg, 2011), quality filtering was performed using fastp (V0.23.1) (Bokulich et al., 2013), data were compared with the reference database [UNITE Database (ITS), https://unite.ut.ee/], and effective data were obtained by removing the chimeric sequences with the vsearch package (V2.16.0, https://github.com/torognes/vsearch) (Edgar et al., 2011). Finally, the optimized data were processed using sequence denoising methods (DADA2/Deblur) in the QIIME2 software (V QIIME2-202202) (Bolyen et al., 2019) to obtain the initial amplicon sequence variant (ASV) sequence and abundance information; subsequent processes (species annotation and phylogenetic relationship construction) were also performed using the QIIME2 software (Walsh et al., 2021). The absolute abundance of ASVs was normalized to 61,466 reads (corresponding to the sample with the fewest sequences) to minimize the effects of sequencing depth on the analysis of community diversity. The FUNGuild database (V1.1) was used to identify putative fungal functional groups (e.g., pathogenic fungi and saprotrophic fungi) (Nguyen et al., 2016).

### Data analysis

To examine the interactive effects of rhizospheric soil microbes and litter on *B. pilosa* in Cd-contaminated soil, a three-way analysis of variance (ANOVA) was conducted to analyze the effects of litter, rhizospheric soil microbes, Cd, and their interactions on various parameters. These parameters included shoot, root, and total biomass and Cd concentration in tissues and soils, as well as the relative abundance of the dominant fungal community at the phylum level. Normality of the data was assessed using the Kolmogorov–Smirnov test, and homogeneity of variance was assessed using Levene’s test. All statistical analyses were performed using the SPSS software (V22.0; IBM Corp., Armonk, NY, USA). Additionally, structural equation modeling (SEM) with the lavaan package (Rosseel, 2012) in R (V4.3.3) was employed to explore the direct and indirect factors (soil N, Ascomycota, and Basidiomycota) that regulate total biomass under rhizosphere soil and litter addition. The fit of the model to the data was determined using the *χ* ^2^ test, goodness-of-fit index (GFI), and root-mean-square error of approximation (RMSEA). The model fit was generally good (*P* > 0.05, GFI close to 1, and RMSEA close to 0).

## Results

### Plant growth

**Figure 2:**
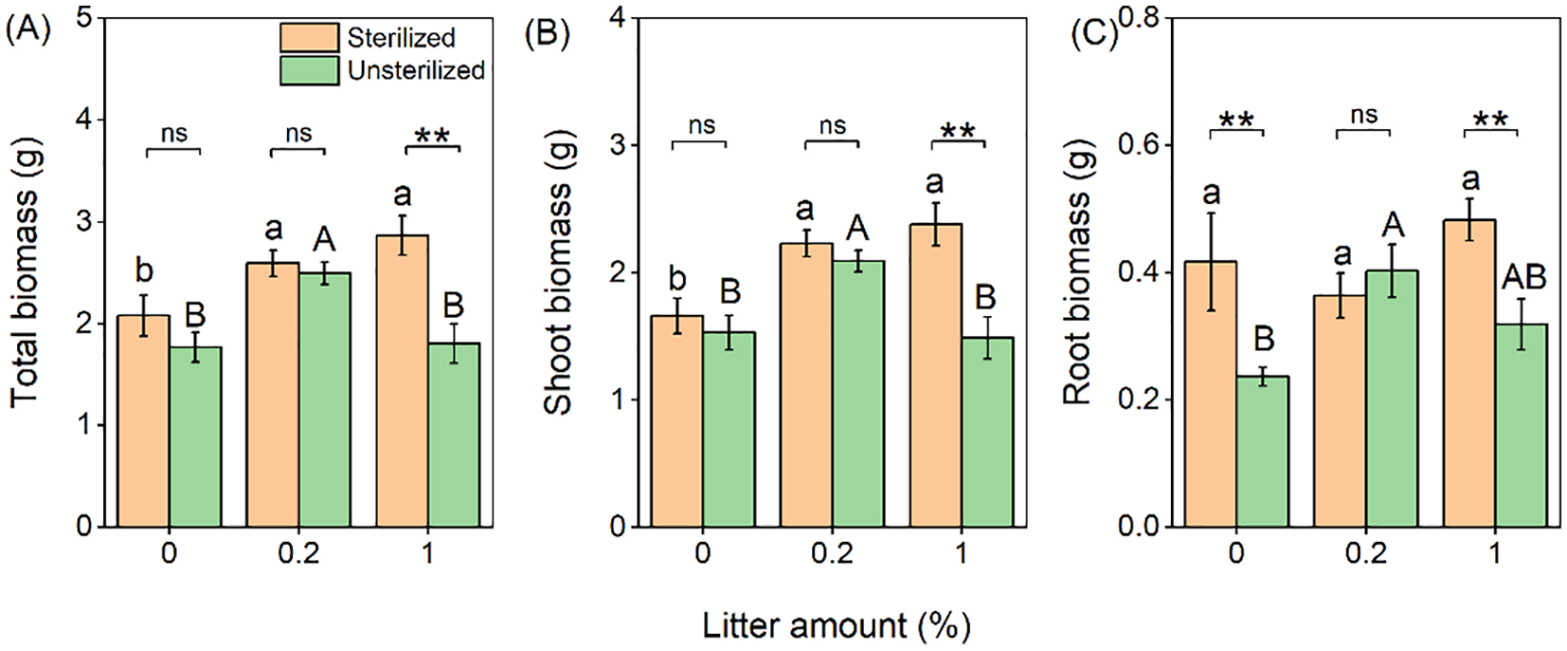
Effects of litter amount (0%, 0.2%, and 1%) and rhizospheric soil microbes (sterilized soil vs. unsterilized soil) on total **(A)**, shoot **(B)**, and root biomass **(C)** of *Bidens pilosa*. Bars and error lines represent mean ± SE. Differences between bars within each litter amount are indicated by the following: ns, non-significant (*P* > 0.05), **P < 0.01. Different lowercase and uppercase letters above the bars indicate the significant differences among different litter amounts under sterilized rhizosphere soil and unsterilized rhizosphere soil, respectively.

The addition of litter significantly increased the total and shoot biomass of *B. pilosa*, by an average of 27% and 28%, respectively (**Table 1**; **Figures 2A, B**). However, no significant differences were observed between the different amounts (0.2% and 1%). Conversely, the presence of unsterilized rhizosphere soil significantly reduced total (−19%), shoot (−18%), and root biomass (−24%) of *B. pilosa* (**Table 1**; **Figures 2A–C**). Furthermore, the negative effect of unsterilized rhizosphere soil on biomass was significantly influenced by litter, with the effect being stronger when 1% litter was applied compared to when 0.2% litter was applied (**Table 1**).

**Table 1:** Results of three-way ANOVAs for the effects of litter (L), rhizospheric soil microbes (M), Cd (Cd) and their interactions on plant growth, tissue Cd, soil parameters, and relative abundance of two dominant fungi phyla.

	Litter (L)	Microbes (M)	Cd	L × M	L × Cd	M × Cd	L × M × Cd
Plant growth							
Shoot biomass	8.42^***^	11.73^***^	0.48	5.00^**^	0.79	1.31	0.11
Root biomass	1.77	9.18^**^	1.47	4.42^*^	3.41^*^	1.68	3.03
Total biomass	7.22^**^	12.97^***^	0.77	4.59^*^	1.40	1.62	0.44
Tissues Cd concentration							
Shoot Cd	5.96^**^	2.92	64.63^***^	1.98	1.61	11.22^***^	0.46
Root Cd	16.73^***^	0.48	72.38^***^	8.04^***^	4.12^*^	0.37	4.57^*^
Soil parameters							
Soil Cd	3.36^*^	<0.01	67.8^***^	5.00^**^	1.27	0.39	1.09
Soil N	1.24	15.25^***^	0.74	5.08^**^	0.85	0.35	5.61^**^
Relative abundance of dominant fungi phyla							
Ascomycota	8.72^***^	1.79	5.92^*^	3.76^*^	3.57^*^	12.78^***^	0.95
Basidiomycota	11.08^***^	140.76^***^	18.19^***^	7.00^**^	5.30^**^	19.36^***^	3.88^*^

*F*-values and significance levels (*P < 0.05, **P < 0.01, and ***P < 0.001) of ANOVAs are given. Values are in bold when P < 0.05.

### Tissue Cd concentrations

**Figure 3:**
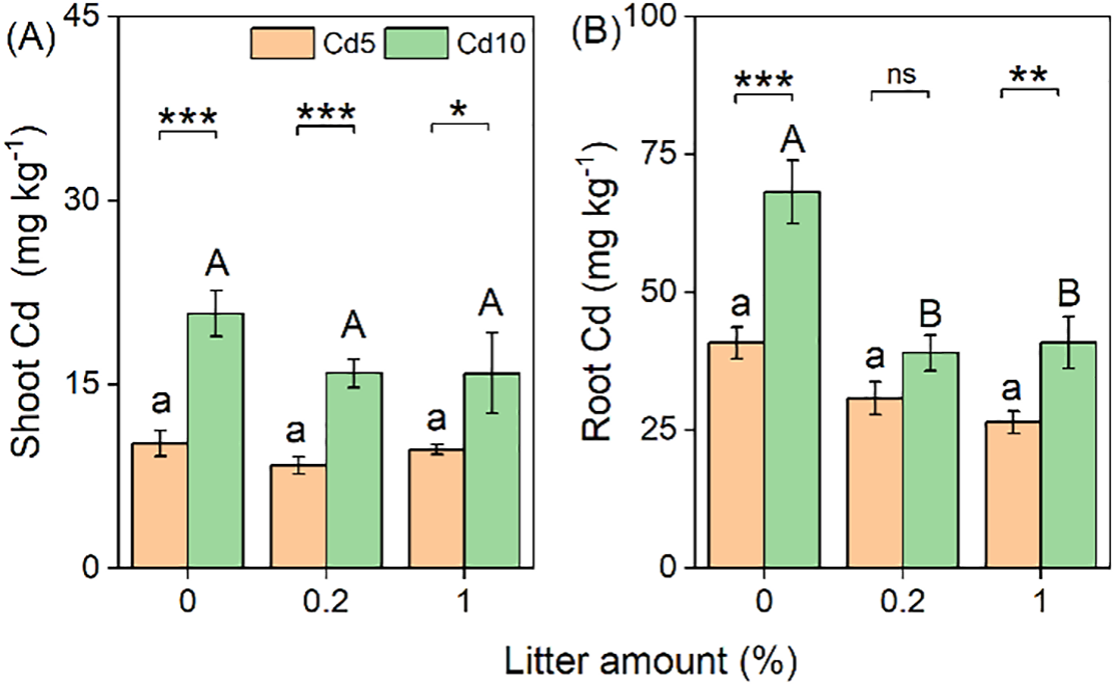
Effects of litter amount (0%, 0.2%, and 1%) and soil Cd (5 mg kg^−1^ and 10 mg kg^−1^) on the Cd concentration in the shoots **(A)** and roots **(B)**. Bars and error lines represent mean ± SE. Differences between bars within each litter amount are indicated by the following: ns, non-significant (*P* > 0.05), *P < 0.05, **P < 0.01, ***P < 0.001. Different lowercase and uppercase letters above the bars indicate the significant differences among different litter amounts under sterilized rhizosphere soil and unsterilized rhizosphere soil, respectively.

The Cd concentrations in both shoots (+75%) and roots (+51%) were significantly higher in the high Cd-contaminated soil than in the low Cd-contaminated soil (**Table 1**; **Figure 3**). However, the Cd concentrations in both shoots and roots significantly decreased with litter addition, by an average of 18% and 37%, respectively (**Table 1**; **Figure 3**).

### Soil parameters

**Figure 4:**
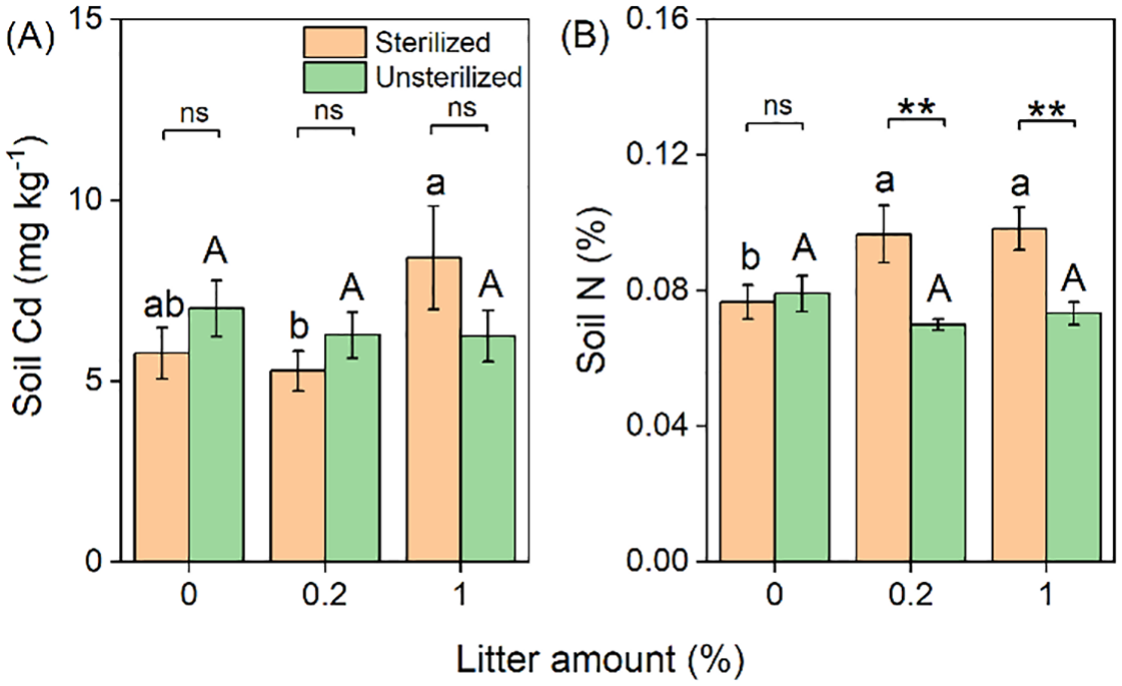
Effects of litter amount (0%, 0.2%, and 1%) and rhizospheric soil microbes (sterilized soil and unsterilized soil) on soil Cd concentration **(A)** and total soil N concentration **(B)**. Bars and error lines represent mean ± SE. Differences between bars within each litter amount are indicated by the following: ns, non-significant (*P* > 0.05), **P < 0.01. Different lowercase and uppercase letters above the bars indicate the significant differences among different litter amounts under sterilized rhizosphere soil and unsterilized rhizosphere soil, respectively.

The concentration of Cd in the soil significantly increased with the addition of litter (**Table 1**; **Figure 4A**). Additionally, the interactive effects of litter and rhizosphere soil were observed, with Cd concentration in soils increasing in sterilized soil but remaining unchanged in unsterilized soil with increasing litter amounts (**Table 1**; **Figure 4A**). Similar effects were observed for soil N (**Table 1**; **Figure 4B**).

### Relative abundances of dominant fungal phyla

**Figure 5:**
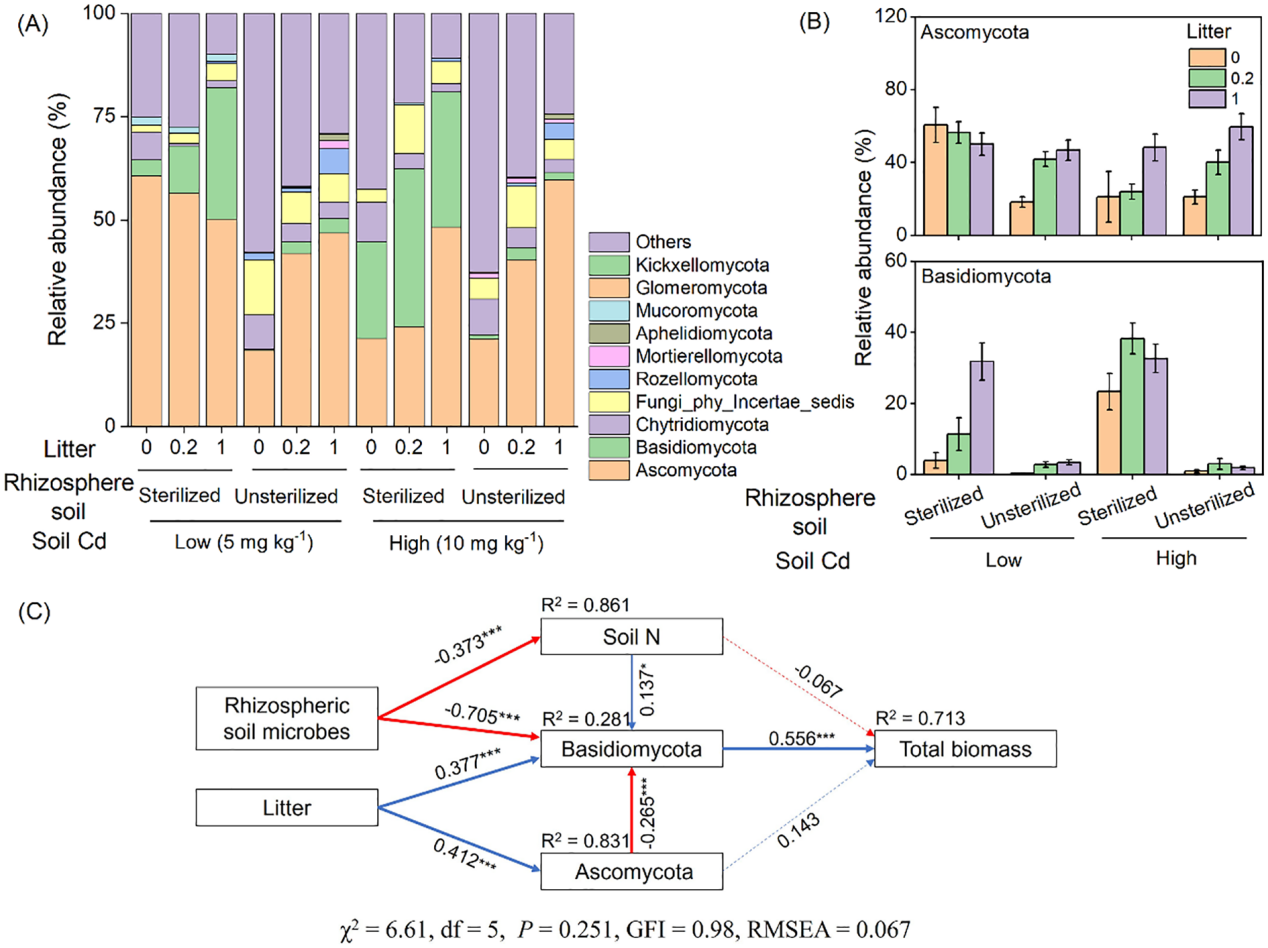
The relative abundance of dominant (top 10) fungi phyla **(A)** and relative abundance of the two dominant phyla Ascomycota and Basidiomycota **(B)**. Structure equation modeling revealed the effects of litter addition and rhizosphere soil on total biomass of *Bidens pilosa* **(C)**. The solid blue arrows indicate significant positive relationships, and the solid red arrows indicate significant negative correlations (*P* < 0.05). The dashed arrows indicate non-significant relationships (*P* > 0.05). Numbers adjacent to the arrows represent standardized path coefficients (^***^ *P* < 0.001). *R* ^2^ values close to the variables indicate the variance explained by the model.

Ascomycota and Basidiomycota were the dominant phyla in the fungal communities, accounting for 41% and 13% of the total sequences, respectively (**Figure 5A**). The addition of litter significantly increased the relative abundance of Ascomycota and Basidiomycota, by an average of 51% and 119%, respectively (**Table 1**; **Figure 5B**). Conversely, the presence of unsterilized rhizosphere soil significantly decreased the relative abundance of Basidiomycota, by an average of 91% (**Table 1**; **Figure 5B**).

### Causal effects revealed by structural equation modeling

Structural equation modeling revealed the direct and indirect effects of soil N, Ascomycota, and Basidiomycota on total biomass under the influence of litter and rhizosphere soil. The rhizosphere soil directly influenced Basidiomycota and indirectly influenced Basidiomycota through soil N, which subsequently influenced total biomass. Litter addition directly influenced Basidiomycota and indirectly influenced Basidiomycota through Ascomycota, ultimately affecting total biomass (**Figure 5C**).

### Putative fungal functional groups

**Figure 6:**
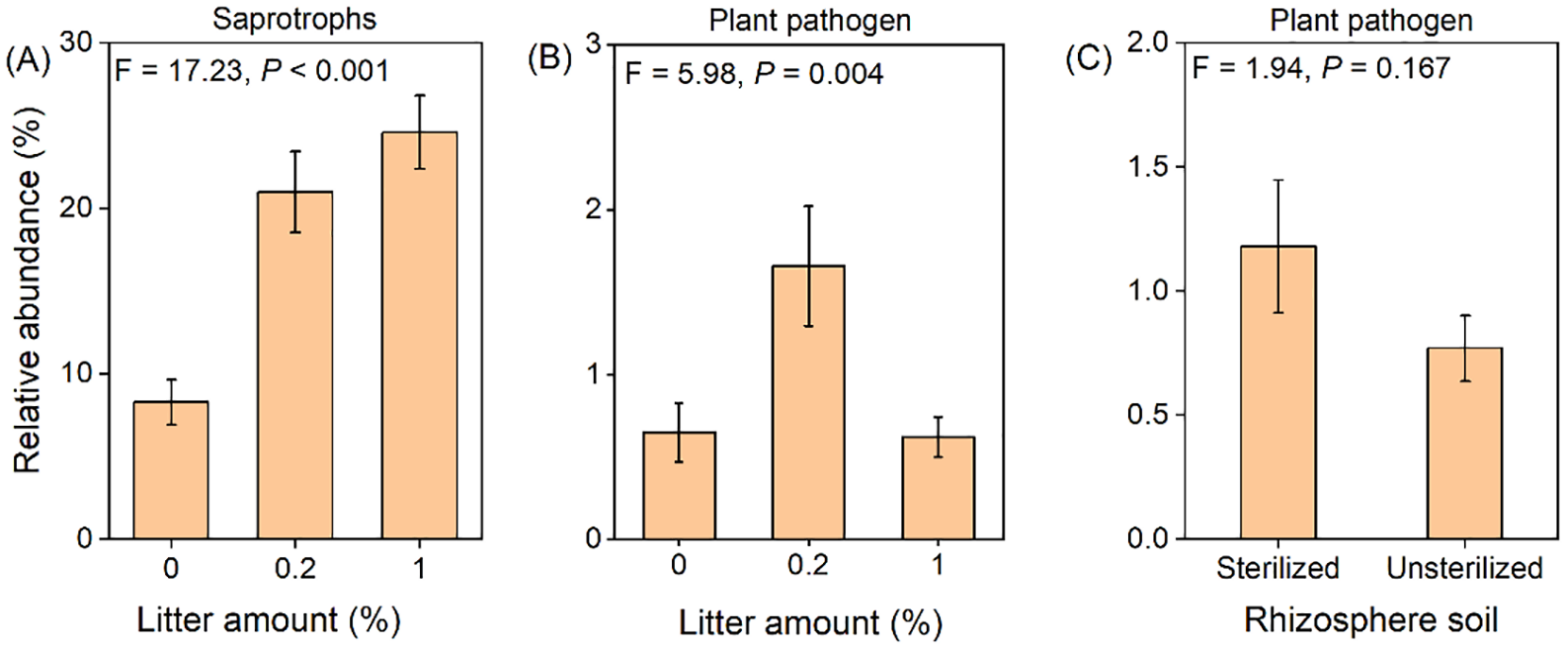
The effect of litter amount (0%, 0.2%, and 1%) on the relative abundance of saprotrophs **(A)** and plant pathogenic fungi **(B)**. Rhizosphere soil (sterilized soil vs. unsterilized soil) on the relative abundance of plant pathogenic fungi **(C)**. Bars and error lines represent mean ± SE. The *F*-values and *P*-values of one-way ANOVA are shown.

The relative abundance of saprotrophs significantly increased with the addition of litter, by an average of 175% (**Figure 6A**). The addition of 0.2% litter significantly increased the abundance of plant pathogenic fungi (+155%), whereas the addition of 1% litter had no significant effect (**Figure 6B**). The rhizosphere soil had no effect on the abundance of plant pathogenic fungi (**Figure 6C**).

## Discussion

Our study revealed that the biomass of *B. pilosa* growing in Cd-contaminated soil was significantly influenced by the application of *B. pilosa* litter and the presence of rhizosphere soil. Specifically, litter application resulted in a significant increase in *B. pilosa* biomass, whereas the unsterilized rhizosphere soil led to a significant decrease. Furthermore, the effects of litter on *B. pilosa* growth were influenced by rhizosphere soil, indicating the importance of soil microbes, particularly fungi, in mediating the performance of *B. pilosa* in Cd-contaminated soil.

The application of *B. pilosa* litter resulted in a notable increase in plant biomass, with no significant difference observed between the two litter amounts (0.2% and 1% litter). This increase was evident in terms of total, shoot, and root biomass, with average increases of 26.8%, 28.2%, and 20.0%, respectively (**Figure 2**). These findings suggest that litter produced by *B. pilosa* exerts positive rather than negative effects on growth. This differs from the potential negative effects of factors such as pathogen accumulation or allelopathic chemicals (Bennett and Klironomos, 2019; Massoni et al., 2021). However, we observed a significant increase in plant pathogens with litter addition at the 0.2% level, which could potentially hinder plant growth. Nonetheless, it appears that the positive effects of litter outweigh the negative effects (Meisner et al., 2012). Litter inputs can influence plant growth by altering nutrient availability (Shen et al., 2016), and previous studies have shown increased nutrient availability due to litter decomposition (Liu et al., 2023, 2022; Wang et al., 2021). Surprisingly, in the present study, the addition of litter did not result in an increase in total soil N, despite the N concentration in the litters (3.08 mg g^−1^) being 33 times higher than that in the soil (0.09 mg g^−1^). One possible explanation is that the amount of litter used (equivalent to a total weight of 0.2% and 1%) might not have been sufficient to significantly affect total soil N, although litter inputs did increase total soil N under sterilized rhizosphere soil (but not under unsterilized) conditions.

Additionally, the presence of litter has been found to supply energy, nutrients, and even microbes to the soil biota, leading to significant changes in the soil microbial composition, including an increase in decomposers (He et al., 2023). Fungi play crucial roles in litter decomposition by actively breaking down the recalcitrant components in plant litter (Veen et al., 2019). Our study demonstrated the significant effects of litter application on the relative abundance of dominant fungal communities at the phylum level (**Table 1**; **Figure 4**) and saprotrophs (**Figure 6A**). Specifically, the relative abundance of the phyla Ascomycota and Basidiomycota, which together accounted for 53% of the total sequences, increased significantly with litter application. Both phyla have been recognized as important decomposers (Dong et al., 2021; Huang et al., 2022; Stursova et al., 2020). Although litter addition had positive effects on both Ascomycota and Basidiomycota, significant negative correlations were observed between them. Furthermore, Basidiomycota contributed more to the total biomass of *B. pilosa* than Ascomycota (**Figure 5C**). This can be attributed to their overlapping substrate resource acquisition; Ascomycota primarily decomposes hemicellulose and cellulose, whereas Basidiomycota decomposes lignin and cellulose (Baldrian, 2017; Manavalan et al., 2015). Thus, Basidiomycota could decompose more complex organic matter, contributing more to increased nutrient availability. Additionally, Basidiomycota has been suggested to contribute more to microbial respiration (Huang et al., 2022) and exhibit higher activities of some enzymes involved in litter decomposition than Ascomycota (Eichlerova et al., 2015). Hence, soil fungi play a crucial role in regulating the effects of litter on the performance of *B. pilosa*.

Our findings indicated that while the application of litter had positive effects on the growth performance of *B. pilosa*, rhizosphere soil exerted negative effects, resulting in a decrease in the biomass of *B. pilosa*. Specifically, rhizosphere soil led to reductions of 18.5%, 24.2%, and 19.5% in the shoot, root, and total biomass of *B. pilosa*, respectively, indicating a negative PSF effect. This aligns with the results of previous studies demonstrating that most plants exhibit negative intraspecific PSF (Bennett and Klironomos, 2019; De Long et al., 2022; Semchenko et al., 2022). The decrease in nutrients, such as N, induced by the rhizosphere soil may have contributed to the growth inhibition of *B. pilosa*, as enriched nutrients typically support plant growth. Additionally, the significantly decreased abundance of Basidiomycota, an important decomposer, may have resulted in the reduced nutrient availability and subsequent growth inhibition of *B. pilosa* (**Figure 5C**). Alternatively, the negative effects of PSF may have been influenced by pathogen accumulation. However, in our study, the abundance of plant pathogenic fungi did not differ in the unsterilized soil and in the sterilized soil (**Figure 6C**).

Furthermore, our results demonstrated that higher soil Cd concentrations did not lead to a decrease in the biomass of *B. pilosa*, despite significantly higher Cd concentrations being detected in the plant tissues under the higher Cd treatment than under the lower Cd treatment. This finding suggests that *B. pilosa* is a highly Cd-tolerant plant, which is consistent with the results of numerous previous studies (Dai et al., 2020; Dou et al., 2019; Li et al., 2023b; Manori et al., 2021). The physiological and molecular mechanisms could explain the Cd tolerance of *B. pilosa*. For example, some physiological characteristics of *B. pilosa*, such as chlorophyll, superoxide dismutase, and peroxidase, were not influenced by Cd (Sun et al., 2009). Additionally, *B. pilosa* could change its protein expression to relieve the oxidative stress caused by Cd (Li et al., 2024). In addition, no interactive effects were observed between litter or rhizospheric soil microbes and soil Cd on the performance of *B. pilosa*. One possible explanation for this is that the dose of Cd used may not have been sufficiently high to induce a negative response in *B. pilosa*, considering its high Cd tolerance. For instance, previous research has shown that the growth of *B. pilosa* was promoted even under a soil Cd concentration of 16 mg kg^−1^ and that it could survive and grow under a soil Cd concentration as high as 100 mg kg^−1^ (Sun et al., 2009). Therefore, in future studies on *B. pilosa* and Cd stress, higher Cd doses should be considered. In addition, one caveat is that our results were obtained from a controlled greenhouse environment with a short duration (approximately 3 months). This indicates that we cannot deeply explain, for example, whether *B. pilosa* has such similar responses in the natural environment. Further studies will take long duration and field experiments into account.

## Conclusion

Our findings indicated that shoot litter addition significantly increased the biomass of *B. pilosa*, whereas unsterilized rhizosphere soil had a significant negative effect on biomass, regardless of the soil Cd concentration. Soil fungi, particularly Basidiomycota, play a crucial role in mediating these effects. These results suggest that litter addition is an effective strategy for mitigating the detrimental effects of Cd toxicity on *B. pilosa*. Although high soil Cd concentrations significantly elevated tissue Cd concentrations, they did not result in a significant reduction in biomass. This could be attributed to the Cd tolerance of *B. pilosa* which is a hyperaccumulator. Additionally, it is possible that the soil Cd dosage used in our study may not have been sufficiently high to cause damage to *B. pilosa*. Future research should consider employing higher soil Cd concentrations, such as 100 mg kg^−1^.

## Data Availability

The original contributions presented in the study are publicly available. This data can be found at http://datadryad.org/stash/share/bbQ3HduSF6E0KWLt85BR-MxuxZK8vmXwrkIDjii1e_8.

## References

[B1] Aldorfova A. Dostalek T. Munzbergova Z. (2022). Effects of soil conditioning, root and shoot litter addition interact to determine the intensity of plant-soil feedback. Oikos 2022, 09025. doi: 10.1111/oik.09025

[B2] Baldrian P. (2017). Forest microbiome: diversity, complexity and dynamics. FEMS Microbiol. Rev. 41, 109–130. doi: 10.1093/femsre/fuw040 27856492

[B3] Bennett J. A. Klironomos J. (2019). Mechanisms of plant-soil feedback: interactions among biotic and abiotic drivers. New Phytol. 222, 91–96. doi: 10.1111/nph.2019.222.issue-1 30451287

[B4] Bennett J. A. Maherali H. Reinhart K. O. Lekberg Y. Hart M. M. Klironomos J. (2017). Plant-soil feedbacks and mycorrhizal type influence temperate forest population dynamics. Science 355, 181–184. doi: 10.1126/science.aai8212 28082590

[B5] Bever J. D. Westover K. M. Antonovics J. (1997). Incorporating the soil community into plant population dynamics: the utility of the feedback approach. J. Ecol. 85, 561–573. doi: 10.2307/2960528

[B6] Bezemer T. M. van der Putten W. H. Martens H. van de Voorde T. F. J. Mulder P. P. J. Kostenko O. (2013). Above- and below-ground herbivory effects on below-ground plant-fungus interactions and plant-soil feedback responses. J. Ecol. 101, 325–333. doi: 10.1111/1365-2745.12045

[B7] Bokulich N. A. Subramanian S. Faith J. J. Gevers D. Gordon J. I. Knight R.. (2013). Quality-filtering vastly improves diversity estimates from Illumina amplicon sequencing. Nat. Methods 10, 57–59. doi: 10.1038/nmeth.2276 23202435 PMC3531572

[B8] Bolyen E. Rideout J. R. Dillon M. R. Bokulich N. A. Abnet C. C. Al-Ghalith G. A.. (2019). Reproducible, interactive, scalable and extensible microbiome data science using QIIME 2. Nat. Biotechnol. 37, 852–857. doi: 10.1038/s41587-019-0209-9 31341288 PMC7015180

[B9] Dai H. Wei S. Skuza L. (2020). Effects of different soil pH and nitrogen fertilizers on *Bidens pilosa* L. Cd accumulation. Environ. Sci. pollut. R. 27, 9403–9409. doi: 10.1007/s11356-019-07579-5 31916155

[B10] De Long J. R. Heinen R. Hannula S. E. Jongen R. Steinauer K. Bezemer T. M. (2022). Plant-litter-soil feedbacks in common grass species are slightly negative and only marginally modified by litter exposed to insect herbivory. Plant Soil 485, 227–244. doi: 10.1007/s11104-022-05590-3

[B11] Dong X. Gao P. Zhou R. Li C. Dun X. Niu X. (2021). Changing characteristics and influencing factors of the soil microbial community during litter decomposition in a mixed *Quercus acutissima* Carruth. and *Robinia pseudoacacia* L. forest in Northern China. Catena 196, 104811. doi: 10.1016/j.catena.2020.104811

[B12] Dou X. Dai H. Skuza L. Wei S. (2019). Bidens pilosa L. hyperaccumulating Cd with different species in soil and the role of EDTA on the hyperaccumulation. Environ. Sci. pollut. R. 26, 25668–25675. doi: 10.1007/s11356-019-05831-6 31267398

[B13] Edgar R. C. Haas B. J. Clemente J. C. Quince C. Knight R. J. B. (2011). UCHIME improves sensitivity and speed of chimera detection. Bioinformatics 27, 2194–2200. doi: 10.1093/bioinformatics/btr381 21700674 PMC3150044

[B14] Ehrenfeld J. G. Ravit B. Elgersma K. (2005). Feedback in the plant-soil system. Annu. Rev. Env. Resour. 30, 75–115. doi: 10.1146/annurev.energy.30.050504.144212

[B15] Eichlerova I. Homolka L. Zifcakova L. Lisa L. Dobiasova P. Baldrian P. (2015). Enzymatic systems involved in decomposition reflects the ecology and taxonomy of saprotrophic fungi. Fungal Ecol. 13, 10–22. doi: 10.1016/j.funeco.2014.08.002

[B16] Eppinga M. B. Molofsky J. (2013). Eco-evolutionary litter feedback as a driver of exotic plant invasion. Perspect. Plant Ecol. 15, 20–31. doi: 10.1016/j.ppees.2012.10.006

[B17] Fetzer J. Moiseev P. Frossard E. Kaiser K. Mayer M. Gavazov K.. (2024). Plant-soil interactions alter nitrogen and phosphorus dynamics in an advancing subarctic treeline. Glob. Change Biol. 30, e17200. doi: 10.1111/gcb.17200 38433308

[B18] Friman J. Karssemeijer P. N. Haller J. de Kreek K. van Loon J. J. A. Dicke M. (2021). Shoot and root insect herbivory change the plant rhizosphere microbiome and affects cabbage-insect interactions through plant-soil feedback. New Phytol. 232, 2475–2490. doi: 10.1111/nph.v232.6 34537968 PMC9291931

[B19] Garcia-Parisi P. A. Omacini M. (2017). Arbuscular mycorrhizal fungi can shift plant-soil feedback of grass-endophyte symbiosis from negative to positive. Plant Soil 419, 13–23. doi: 10.1007/s11104-017-3216-y

[B20] Haider F. U. Liqun C. Coulter J. A. Cheema S. A. Wu J. Zhang R.. (2021). Cadmium toxicity in plants: Impacts and remediation strategies. Ecotox. Environ. Safe. 211, 111887. doi: 10.1016/j.ecoenv.2020.111887 33450535

[B21] He Y. Jia B. Wei C. Fan F. Wilschut R. A. Lu X. (2023). Leaf litter presence in the non-growing season prolongs plant legacy effects on soil fungal communities and succeeding plant growth. J. Ecol. 111, 1997–2009. doi: 10.1111/1365-2745.14157

[B22] Huang C. Wu X. Liu X. Fang Y. Liu L. Wu C. (2022). Functional fungal communities dominate wood decomposition and are modified by wood traits in a subtropical forest. Sci. Total Environ. 806, 151377. doi: 10.1016/j.scitotenv.2021.151377 34740660

[B23] Idbella M. Bonanomi G. De Filippis F. Foscari A. Zotti M. Abd-ElGawad A. M.. (2024). Negative plant-soil feedback in *Arabidopsis thaliana*: Disentangling the effects of soil chemistry, microbiome, and extracellular self-DNA. Microbiol. Res. 281, 127634. doi: 10.1016/j.micres.2024.127634 38308902

[B24] Jing J. Cong W.-F. Bezemer T. M. (2022). Legacies at work: plant-soil-microbiome interactions underpinning agricultural sustainability. Trends Plant Sci. 27, 781–792. doi: 10.1016/j.tplants.2022.05.007 35701291

[B25] Kadowaki K. (2024). Forest tree community ecology and plant-soil feedback: Theory and evidence. Ecol. Res. 39, 257–272. doi: 10.1111/1440-1703.12445

[B26] Li Y. Rahman S. U. Qiu Z. Shahzad S. M. Nawaz M. F. Huang J.. (2023a). Toxic effects of cadmium on the physiological and biochemical attributes of plants, and phytoremediation strategies: A review. Environ. pollut. 325, 121433. doi: 10.1016/j.envpol.2023.121433 36907241

[B27] Li Y. Shi X. Tan W. Ling Q. Pei F. Luo S.. (2023b). Metagenomics combined with metabolomics reveals the effect of Enterobacter sp. inoculation on the rhizosphere microenvironment of *Bidens pilosa* L. @ in heavy metal contaminated soil. J. Hazard. Mater. 458, 132033. doi: 10.1016/j.jhazmat.2023.132033 37453352

[B28] Li Y. Shi X. Xu J. Huang X. Feng J. Huang Y.. (2024). Proteomics-based analysis on the stress response mechanism of *Bidens pilosa* L. under cadmium exposure. J. Hazard. Mater. 462, 132761. doi: 10.1016/j.jhazmat.2023.132761 37837780

[B29] Liu J. Wang J. Morreale S. J. Schneider R. L. Li Z. Wu G.-L. (2023). Contributions of plant litter to soil microbial activity improvement and soil nutrient enhancement along with herb and shrub colonization expansions in an arid sandy land. Catena 227, 107098. doi: 10.1016/j.catena.2023.107098

[B30] Liu S. Yang R. Peng X. Hou C. Ma J. Guo J. (2022). Contributions of plant litter decomposition to soil nutrients in ecological tea gardens. Agriculture-Basel 12, 957. doi: 10.3390/agriculture12070957

[B31] Magoč T. Salzberg S. L. (2011). FLASH: fast length adjustment of short reads to improve genome assemblies. Bioinformatics 27, 2957–2963. doi: 10.1093/bioinformatics/btr507 21903629 PMC3198573

[B32] Manavalan T. Manavalan A. Heese K. (2015). Characterization of lignocellulolytic enzymes from white-rot fungi. Curr. Microbiol. 70, 485–498. doi: 10.1007/s00284-014-0743-0 25487116

[B33] Manori S. Shah V. Soni V. Dutta K. Daverey A. (2021). Phytoremediation of cadmium-contaminated soil by *Bidens pilosa* L.: impact of pine needle biochar amendment. Environ. Sci. pollut. R. 28, 58872–58884. doi: 10.1007/s11356-021-12953-3 33599932

[B34] Massoni J. Bortfeld-Miller M. Widmer A. Vorholt J. A. (2021). Capacity of soil bacteria to reach the phyllosphere and convergence of floral communities despite soil microbiota variation. P. Natl. Acad. Sci. 118, e2100150118. doi: 10.1073/pnas.2100150118 PMC8521660 34620708

[B35] Mazurek R. Kowalska J. Gasiorek M. Zadrozny P. Jozefowska A. Zaleski T.. (2017). Assessment of heavy metals contamination in surface layers of Roztocze National Park forest soils (SE Poland) by indices of pollution. Chemosphere 168, 839–850. doi: 10.1016/j.chemosphere.2016.10.126 27829506

[B36] Mazzoleni S. Bonanomi G. Incerti G. Chiusano M. L. Termolino P. Mingo A.. (2015). Inhibitory and toxic effects of extracellular self-DNA in litter: a mechanism for negative plant-soil feedbacks? New Phytol. 205, 1195–1210. doi: 10.1111/nph.2015.205.issue-3 25354164

[B37] Meisner A. de Boer W. Cornelissen J. H. C. van der Putten W. H. (2012). Reciprocal effects of litter from exotic and congeneric native plant species via soil nutrients. PloS One 7, e31596. doi: 10.1371/journal.pone.0031596 22359604 PMC3281088

[B38] Nguyen N. H. Song Z. Bates S. T. Branco S. Tedersoo L. Menke J.. (2016). FUNGuild: An open annotation tool for parsing fungal community datasets by ecological guild. Fungal Ecol. 20, 241–248. doi: 10.1016/j.funeco.2015.06.006

[B39] Palansooriya K. N. Shaheen S. M. Chen S. S. Tsang D. C. W. Hashimoto Y. Hou D.. (2020). Soil amendments for immobilization of potentially toxic elements in contaminated soils: A critical review. Environ. Int. 134, 105046. doi: 10.1016/j.envint.2019.105046 31731004

[B40] Rosseel Y. (2012). lavaan: an R package for structural equation modeling. J. Stat. Software 48, 1–36. doi: 10.18637/jss.v048.i02

[B41] Semchenko M. Barry K. E. de Vries F. T. Mommer L. Moora M. Macia-Vicente J. G. (2022). Deciphering the role of specialist and generalist plant-microbial interactions as drivers of plant-soil feedback. New Phytol. 234, 1929–1944. doi: 10.1111/nph.v234.6 35338649

[B42] Shen Y. Chen W. Yang G. Yang X. Liu N. Sun X.. (2016). Can litter addition mediate plant productivity responses to increased precipitation and nitrogen deposition in a typical steppe? Ecol. Res. 31, 579–587. doi: 10.1007/s11284-016-1368-5

[B43] Stursova M. Snajdr J. Koukol O. Tlaskal V. Cajthaml T. Baldrian P. (2020). Long-term decomposition of litter in the montane forest and the definition of fungal traits in the successional space. Fungal Ecol. 46, 100913. doi: 10.1016/j.funeco.2020.100913

[B44] Sun J. Rutherford S. Saif Ullah M. Ullah I. Javed Q. Rasool G.. (2022). Plant-soil feedback during biological invasions: effect of litter decomposition from an invasive plant (*Sphagneticola trilobata*) on its native congener (*S. calendulacea*). J. Plant Ecol. 15, 610–624. doi: 10.1093/jpe/rtab095

[B45] Sun Y. Zhou Q. Wang L. Liu W. (2009). Cadmium tolerance and accumulation characteristics of *Bidens pilosa* L. as a potential Cd-hyperaccumulator. J. Hazard. Mater. 161, 808–814. doi: 10.1016/j.jhazmat.2008.04.030 18513866

[B46] Teste F. P. Kardol P. Turner B. L. Wardle D. A. Zemunik G. Renton M.. (2017). Plant-soil feedback and the maintenance of diversity in Mediterranean-climate shrublands. Science 355, 173–176. doi: 10.1126/science.aai8291 28082588

[B47] van der Putten W. H. Bardgett R. D. Bever J. D. Bezemer T. M. Casper B. B. Fukami T.. (2013). Plant-soil feedbacks: the past, the present and future challenges. J. Ecol. 101, 265–276. doi: 10.1111/jec.2013.101.issue-2

[B48] Veen C. Fry E. Hooven F. Kardol P. Morriën E. De Long J. (2019). The role of plant litter in driving plant-soil feedbacks. Front. Environ. Sci. 7, 168. doi: 10.3389/fenvs.2019.00168

[B49] Voriskova J. Baldrian P. (2013). Fungal community on decomposing leaf litter undergoes rapid successional changes. Isme J. 7, 477–486. doi: 10.1038/ismej.2012.116 23051693 PMC3578564

[B50] Walsh C. M. Becker-Uncapher I. Carlson M. Fierer N. (2021). Variable influences of soil and seed-associated bacterial communities on the assembly of seedling microbiomes. ISME J. 15, 2748–2762. doi: 10.1038/s41396-021-00967-1 33782567 PMC8397733

[B51] Wang X.-X. Hoffland E. Mommer L. Feng G. Kuyper T. W. (2019). Maize varieties can strengthen positive plant-soil feedback through beneficial arbuscular mycorrhizal fungal mutualists. Mycorrhiza 29, 251–261. doi: 10.1007/s00572-019-00885-3 30919070

[B52] Wang J. Xu B. Wu Y. Gao J. Shi F. Wu N. (2021). Effect of inflorescence litter from distinct species and life forms on soil nutrients and microbial biomass in the eastern Tibetan Plateau. Glob. Ecol. Conserv. 31, e01825. doi: 10.1016/j.gecco.2021.e01825

[B53] Wang X. Zheng W. L. Yuan H. M. van Kleunen M. Yu F. H. Li M. H. (2024). Biochar produced from diverse invasive species improves remediation of cadmium-contaminated soils. Biol. Invasions 26, 2595–2606. doi: 10.1007/s10530-024-03332-3

[B54] Wu C. Zhang X. Xie C. Yue C. Li H. Wang J.. (2019). Heavy metal pollution characteristics and ecological risk assessment of 4 greening types soils in Luqiao, Taizhou. J. Zhejiang For. Sci. Technol. 39, 38–44. doi: 10.3969/j.issn.1001-3776.2019.05.006

[B55] Zhang X. Gu P. Liu X. Huang X. Wang J. Zhang S.. (2021). Effect of crop straw biochars on the remediation of Cd-contaminated farmland soil by hyperaccumulator Bidens pilosa L. Ecotox. Environ. Safe. 219, 112332. doi: 10.1016/j.ecoenv.2021.112332 34044313

[B56] Zhang P. Li B. Wu J. Hu S. (2019). Invasive plants differentially affect soil biota through litter and rhizosphere pathways: a meta-analysis. Ecol. Lett. 22, 200–210. doi: 10.1111/ele.2019.22.issue-1 30460738

[B57] Zhang N. Li Y. Wubet T. Bruelheide H. Liang Y. Purahong W.. (2018). Tree species richness and fungi in freshly fallen leaf litter: Unique patterns of fungal species composition and their implications for enzymatic decomposition. Soil Biol. Biochem. 127, 120–126. doi: 10.1016/j.soilbio.2018.09.023

[B58] Zheng W. L. Wang Y. F. Mo J. Y. Zeng P. Chen J. Y. Sun C. L. (2023). Effects of biochar application and nutrient fluctuation on the growth, and cadmium and nutrient uptake of *Trifolium repens* with different planting densities in Cd-contaminated soils. Front. Plant Sci. 14, 1269082. doi: 10.3389/fpls.2023.1269082 37799556 PMC10548119

[B59] Zotti M. Bonanomi G. Saulino L. Allevato E. Saracino A. Mazzoleni S.. (2023). Shifts of leaf litter-induced plant-soil feedback from negative to positive driven by ectomycorrhizal symbiosis between *Quercus ilex* and *Pisolithus arrhizus* . Microorganisms 11, 1394. doi: 10.3390/microorganisms11061394 37374896 PMC10300854

